# A Pilot Study of Extended Adjuvant Therapy with Metronomic Docetaxel for Patients with Operable Triple-Negative Breast Cancer

**DOI:** 10.31557/APJCP.2020.21.3.749

**Published:** 2020-03

**Authors:** Bader A Abdelmaksoud, Abdelmotaleb Mohamed, Mostafa M Toam

**Affiliations:** 1 *Department of Clinical Oncology and Nuclear Medicine, Faculty of Medicine, Zagazig university, Zagazig, Egypt. *

**Keywords:** Metronomic chemotherapy, docetaxel, TNBC, extended adjuvant therapy

## Abstract

**Background:**

Triple-negative breast(TNBC) cancer is a molecular subtype of breast cancer with poor prognosis and did not get approved targeted therapy till now. In the last years, metronomic chemotherapy (mCTH) was investigated to improve treatment outcomes in TNBC patients both in early and metastatic setting due to its anti-angiogenic and immune-stimulatory mechanisms. The aim of this study is to evaluate the efficacy and safety of extended adjuvant chemotherapy with metronomic docetaxel for patients with operable TNBC.

**Methods:**

31 women with clinically and pathologically proved operable TNBC, either node-negative or node-positive with tumor size ≥ 0,5 cm were enrolled after finishing the primary standard of care treatment. The patients were subjected to extended adjuvant therapy for 6 months with metronomic low dose docetaxel with starting dose of 15mg/m^2^ in weekly bases for 4 weeks then the dose was escalated to 20 mg/m^2 ^once per week if there were no side effects.

**Results:**

After a median follow up of 36 months (range 6-52), 24 patients (77.4%) were still alive. During the period of follow-up, 12 patients (38.7%) showed disease relapse and 19(61.3%) cases remain free of the disease. The estimated mean of DFS in our study was 38.26 months (95%CI; 31.87 – 44.65) with 2 and 3 years DFS rate of 70.5 % and 56.4% respectively while the estimated mean of OS was 43.75 months (95% CI; 38.35 – 49.16) with 2 and 3 years OS rates 83.3% and 78.1% respectively, Generally the treatment was tolerated with mild to moderate hematological and non hematological adverse events, all are grade 1,2 and treatment-related deaths were not observed.

**Conclusion:**

Extended adjuvant treatment for 6 months with metronomic docetaxel after the primary standard of care therapy was tolerated and has an encouraging survival benefit in patients with operable TNBC and these results need further evaluation in randomized control studies.

## Introduction

Generally, breast cancer (BC) is a highly heterogeneous disease at both clinical and molecular settings, Triple-negative breast cancer (TNBC) accounts for around 20% of the all BC molecular subtypes and has a very worse prognosis with short overall survival (Fragomeni et al., 2018). After the primary treatment of TNBC, the median time for tumor relapse is approximately 18-20 months with subsequent less than 2 years overall survival after relapse (Bauer et al., 2017). 

In comparison to other BC subtypes, TNBC cases are prone to develop visceral metastases more common than bone metastases and occur in younger age groups. Although there is a relative value of chemotherapy in these cases and a large number of clinical studies, no optimal neo/adjuvant chemotherapy regimen has been identified (Locatelliet al., 2017). 

A novel approach to improve disease-free survival and subsequent overall survival of TNBC patients is the use of extended adjuvant treatment with metronomic chemotherapy (mCTH). In (mCTH), the chemotherapeutic drug can cause modulation in the cancer cell microenvironment and disruption of the tumor angiogenesis, this could be achieved by administering chemotherapeutic agent in low doses on weekly or even daily bases without a prolonged interval (Kareva et al., 2015). Various preclinical and clinical studies have shown metronomic chemotherapy might show alternative anti-neoplastic effects of the drugs in addition to its direct cytotoxic effect (Cazzaniga etal., 2019; Abdelmaksoud et al., 2019). The main objective of mCTH is to achieve a comparable or good efficacy along with tolerability and safety compared to standard conventional chemotherapy, Also, it is considered by some authors as an attractive novel approaches with additional anti-angiogenic effects with lesser toxicities compared to the standard chemotherapy (Banys-Paluchowski et al., 2016). 

In TNBC, there are many clinical trials investigating the role of extended adjuvant therapy with metronomic chemotherapy after the primary standard treatment (LIuch et al.,2010). In the preclinical setting, Di Desidero and colleagues investigated the potential effect of combination of metronomic low dose of topotecan with pazopanib in primary or metastatic triple-negative breast cancer cell lines, and their conclusion was that combination needs further evaluation being a potential therapeutic option for that type of breast cancer (Di Desidero et al., 2015). In clinical studies, there are many studies investigated the role of mCTH in patients with non-metastatic TNBC and will be mentioned in the discussion section (Rabanal et al., 2017). 

Docetaxel has been recognized both in vivo and in vitro studies as an active agent that can exhibit a metronomic activitiy (Kamat et al., 2007). The efficacy of taxanes both neoadjuvant and adjuvant settings in TNBC was established and still part of the standard of care in the management of this aggressive subtype of breast cancer. Therefore, we hypothesized that patients with TNBC might achieve a survival benefit from mCTH with docetaxel. 

In this study, we evaluated the efficacy and toxicity of a novel metronomic extended adjuvant regimen in patients with early TNBC. As far as the authors know, this is the first study of a true weekly metronomic regimen with docetaxel in TNBC.

## Materials and Methods

*Patients Eligibility*

This study was conducted at the department of clinical oncology and nuclear medicine, Zagazig university hospitals from April 2015 to February 2018 where 31 women with clinically and pathologically proved operable triple-negative breast cancer, either node-negative or node-positive with tumor size≥0.5cm were eligible for entry of this study, all patients were followed till August 2019. Other inclusion criteria are: age 18-70 years, adequate bone marrow and organ functions, and ECOG performance status (PS) 1-2. Patient was excluded if there is distant metastasis or other active malignancy in the body elsewhere, previous docetaxel in the primary adjuvant treatment, other, also, patient who did not wish to continue on the treatment plan had permission to be excluded. Informed written consent from all patients.

*Study design and Ttreatment plan*

This is a prospective, single-institution, phase II single-arm study. The protocol of the study was approved by the ethical committee in the department of clinical oncology. The patients met above inclusion criteria and had received standard adjuvant treatment either anthracycline or taxane based chemotherapy with adjuvant radiotherapy if indicated were subjected to extended adjuvant therapy with metronomic low dose docetaxel with starting dose of 15mg/m^2^ in weekly bases for 4 weeks then the dose was escalated to 20 mg/m^2^ once per week if there was no neutropenia. Before docetaxel administration, antiemetic, H1-blocker, and 4mg dexamethasone were given as a pre-medications to prevent vomiting and hypersensitivity reaction, then docetaxel administrated as an intravenous infusion in 250 ml dextrose 5% over 60 minutes. The treatment was continued for 6 months if there was intractable toxicity or development of local or distant metastasis.

*Patients assessment and Treatment follow-up*

During the treatment, patients were monitored weekly by general and laboratory examinations for bone marrow and organ function reserves to confirm the tolerability of the treatment. Detection of disease progression was monitored every 3 months during and after the treatment by general physical examination, chest x-ray, abdominopelvic ultrasound, and mammography. Computed tomography (CT) was ordered if there were any suspicious lesions in ultrasound or x-ray, isotopic bone scan ordered only if there were symptoms suggestive of bone metastasis or increased serum alkaline phosphatase, otherwise annually. Treatment-related adverse effects were graded according to the common terminology criteria of adverse events (NCI-CTC v3).

*Endpoints*

The primary endpoints of this study were treatment tolerability and disease-free survival while the secondary endpoint was overall survival.

*Statistical analysis*

All statistics were performed using SPSS 22.0 for windows (SPSS Inc., Chicago, IL, USA) and MedCalc windows (MedCalc Software bvba 13, Ostend, Belgium). Disease-Free Survival (DFS) was calculated as the time from date of surgery to date of 1st disease relapse or the most recent follow-up that patient was known as free from the disease while Overall Survival (OS) was calculated as the time from diagnosis of the disease to the death of the patient or for the most recent follow-up (censored).

**Table 1:** Patients and Tumor Characteristics

Characteristics		All patients (N=31)			
		No.		%	
Age					
Mean ± SD		48.70		±10.63	
Median (Range)		47		(28 – 69)	
<35 years		3		9.7%	
≥35 years		28		90.3%	
Menopausal status					
Premenopausal		13		41.9%	
Postmenopausal		18		58.1%	
Perfomance status					
ECOG 0		19		61.3%	
ECOG 1		9		29%	
ECOG 2		3		9.7%	
Histology					
IDC		28		90.3%	
Others		3		9.7%	
Grade					
Grade 1		2		6.5%	
Grade 2		14		45.2%	
Grade 3		15		48.4%	
Tumor stage					
T 1		5		16.1%	
T 2		20		64.5%	
T 3		6		19.4%	
Nodal status					
N 0		14		45.2%	
N 1		11		35.5%	
N 2		6		19.4%	
Surgery					
BCS		5		16.1%	
MRM		26		83.9%	
Adjuvant radiotherapy				
No	15		48.4%	
Yes	16		51.6%	
Adjuvant chemotherapy				
6 FAC	7		22.6%	
6 FEC 100	1		3.2%	
4 AC-4T	20		64.5%	
3 FEC-4T	3		9.7%	
ExAj duration (months)				
Mean ± SD	5.74		±0.81	
Median (Range)	6		(3 – 6)	
3 months	2		6.5%	
4 months	1		3.2%	
6 months	28		90.3%	

ExAj, extended adjuvant; ICD, invasive duct carcinoma.

**Table 2.** Treatment Outcome

Outcome	All patients (N=31)	
	No.	%
Follow-up duration (months)		
Mean ± SD	32.09	±12.81
Median (Range)	36	(6 – 52)
Relapse		
Absent	19	61.3%
Present	12	38.7%
Disease Free Survival (DFS)		
Mean DFS	38.26 months	
(95%CI)	(31.87 – 44.65)	
12 month DFS	83.9%	
24 month DFS	70.5%	
36 month DFS	56.4%	
48 month DFS	56.4%	
Mortality		
Alive	24	77.4%
Died	7	22.6%
Overall Survival (OS)		
Mean OS	43.75 months	
(95%CI)	(38.35 – 49.16)	
12 month OS	90%	
24 month OS	83.3%	
36 month OS	78.1%	
48 month OS	68.4%	

Continuous variables were expressed as mean (95%CI); Categorical variables were expressed as number (percentage).

**Figure 1:**
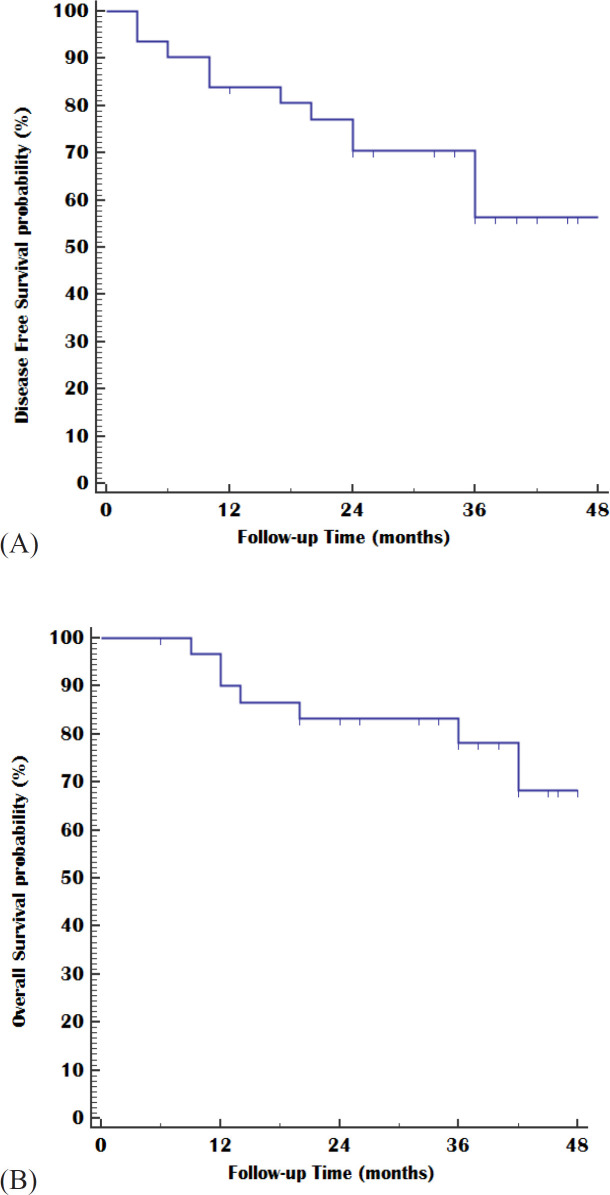
Kaplan-Meier Plot; (A) Disease-Free Survival and (B) Overall Survival

**Table 3:** Treatment-related Adverse Effects (AEs)

Toxicity	Grade		
	Grade 1,2 N (%)	Grade 3 N (%)	Grade 4 N (%)
1- Hematological			
Neutropenia	8 (25 %)	3 (1%)	-
Anemia	10 (32%)	8 (25%)	2 (0.6%)
Thrombocytopenia	3 (1%)	-	-
2- Non-hematological			
Hepatotoxicity	10 (30%)	-	-
Nephrotoxicity	3 (1%)	-	-
Diarrhea	3 (1%)	-	-
Pleural effusion	12 (38%)	-	--
Peripheral edema	2 (0.6%)	-	-

## Results

*Basic characteristics*

31 patients with clinically and histologically confirmed operable triple-negative breast cancer were enrolled in this research. Table 1 shows patients and tumor characteristics of studied patients, the median age of the patients was 47 (range, 28-69) 3 patients (9.7%) were ≤ 35 years and 28 (90.3%) were ≥35 years. 13 patients (41.9 %) were premenopausal, 18 patients (58.1%) were postmenopausal. The majority of cases had ECOG PS 0. Also, the majority of patients had invasive duct carcinoma histology (IDC) (90.3%) and grade 3 (48.4%). Regarding the tumor size and nodal status, most patients (64.5%) had T2 stage and (45.2%) had N0. In this study, 26 patients (83.4%) underwent modified radical mastectomy (MRM) while only 5 cases (16.1) underwent breast conservative surgery (BCS). All patients received adjuvant chemotherapy, the majority of them (64.5 %) received taxane-anthracyclin based regimens. Adjuvant radiotherapy was applied to 16 (51.6%) of patients. The planned duration of extended adjuvant treatment was 6 months, however, only 28 patients (90.3%) completed the planned duration and 3 cases (9.7%) did not continue due to disease relapse.

*Treatment outcome*

Treatment outcome of extended adjuvant treatment with docetaxel for 31 patients with operable triple-negative breast cancer was shown in Table 2. After a median follow-up of 36 months (range 6-52), 24 patients (77.4%) of 31 cases were still alive. During the period of follow-up, 12 patients (38.7%) showed disease relapse and 19 (61.3%) cases remain free of the disease till the end of the follow-up period. The estimated mean of DFS was 38.26 months (95%CI; 31.87 – 44.65) with 2,3, and 4 survival rate were 83.9%,70,5%, and 56.4% respectively. the median DFS not reached, while the estimated mean of OS was 43.75 months (95%CI; 38.35 – 49.16) with 2,3, and 4 OS rate 83.3%,78.1%, and 68.4% respectively. The median OS not reached also, Figure 1 showed the Kaplan-Meier survival curves of both disease-free survival and overall survival .

Regarding the treatment-related toxicities, generally the treatment was tolerated with mild to moderate hematological side effects but there are non-hematological adverse events, all are grade 1, 2 and treatment-related deaths were not observed. Grade 1, 2 anemia is the most common hematological toxicity while pleural effusion is the most frequent non-hematological side effects, Grade 3 neutropenia observed only in 3 (1%) while Grade 3, 4 anemia was observed in 8 (25%) and 2 (0.6%) respectively. For both hematological and non hematological toxicities no dose reduction or interruption required and all side effects were easily manageable. Summary of all treatment-related adverse effects are presented in Discussion

TNBC is a heterogeneous subtype of breast cancer characterized by several molecular variants differed in their natural history and may need different options of treatment. Till now, taxanes and anthracyclines-based therapy is the standard of care regimens if chemotherapy is indicated (Alice et al., 2019). Compared to hormone receptor-positive breast cancer, the duration of adjuvant treatment in TNBC is still an unanswered question, therefore, there are several studies investigating different regimens with different treatment duration (Early Breast Cancer Trialists’ Collaborative Group, 1998).

In the last decade, mCTH showed a promising activity through its anti-angiogenic and immune-stimulatory mechanisms and so, researchers considered TNBC as a very good candidate for this novel regimen (André et al., 2014; Kerbel et al., 2004) . To improve the treatment outcome in TNBC, the researchers tried to investigate the role of extended adjuvant therapy after the primary standard chemotherapy (Locatelliet al., 2017). Several drugs were investigated in this era of research in both phase II and phase III trials, capecitabine was one of the most investigated drugs in maintenance therapy in TNBC. The largest phase III trials that investigated the extended adjuvant mCTH in TNBC are CIBOMA 2004-01/GEICAM 2003-11 and SYSCBS-001 trials. In CIBOMA trial, the efficacy and safety of maintenance therapy of capecitabine were evaluated after primary treatment in patients with early stages TNBC (Martín et al., 2018), while in SYSCBS-001, capecitabine was evaluated as in CIBOMA studies but with different dose and duration Zhong-yu., NCT01112826). Also, the efficacy of extended therapy with capecitabine was evaluated in a famous phase III trial, the CREATE-X trial that showed a significant improvement in survival in patients with TNBC with a residual disease after neoadjuvant therapy (Masuda et al., 2017). 

Docetaxel exhibit significant activity in management of TNBC in both neoadjuvant and adjuvant settings either alone or in combination with other drugs (Ohno et al., 2013). In this study, extended adjuvant treatment with a metronomic low dose of docetaxel was used for patients with operable TNBC after the primary standard adjuvant therapy. 

To our Knowledge, this is the first study that evaluated the safety and efficacy of extended adjuvant chemotherapy with metronomic docetaxel in TNBC. Our hypothesis based on the results of the studies that demonstrated good efficacy and tolerability of metronomic docetaxel in other solid tumors. In lung cancer, Yokoi et al underwent a pilot study to evaluate the toxicity and efficacy of low-dose docetaxel for patient with previously treated non-small cell lung and their conclusion that metronomic regimen was well tolerated and active, but they recommended further investigations (Yokoi et al., 2012). In breast cancer, Young et al investigated the role of metronomic docetaxel combined with capecitabine in patients with metastatic breast cancer and they concluded that metronomic docetaxel combined with capecitabine chemotherapy can produce a significant anticancer effect, with predictable toxicity (Young et al., 2012). 

The preliminary results of our study demonstrated encouraging effects with acceptable toxicities, the estimated mean of DFS in our study was 38.26 months (95% CI; 31.87 – 44.65) with 2 and 3 years DFS rate 70.5 % and 56.4% respectively but the median rate was not reached and the estimated mean of OS was 43.75 months (95%CI; 38.35 – 49.16) with 2 and 3 years OS rates 83.3% and 78.1% respectively, the median OS was not reached also.

Compared to the obtained results of other studies conducted in our country that evaluated the extended adjuvant therapy in TNBC, Nasr and colleagues investigated the safety and efficac of metronomic methotrexate and Cyclophosphamide given for 1 year after the standard adjuvant therapy for cases with TNBC in an attempt to improve the disease-free survival of such patients (Nasr et al., 2015), the median DFS and OS in their results were 28 and 37 months respectively that appeared less than that obtained in our study and his conclusion was that metronomic therapy was well tolerated and achieved significant improvement in both DFS and OS. In another study conducted by Shawky and Galal, 19 patients with operable TNBC were subjected to receive metronomic capecitabine therapy (650 mg/m^2^, twice every day) for one year after finishing the standard adjuvant treatment (Shawky et al., 2014), in their results, the mean DFS was 41.7 months (95% CI; 36.5–46.9) and The 2 and 3 years DFS rates were 88.8% and 82.05%, respectively, that was at some extent the same as that obtained in our study, although the median follow-up period and number of patients is less than that in our research. Also, Alagizy et al underwent another study as that of Shawky study for the same purpose but with different dose and duration schedule, they used 6 mounhs of metronomic capecitabine 500 mg two times by per-day after recieving 6 cycles of adjuvant chemotherapy with FEC100 with or without adjuvant radiotherapy, the mean DFS and mean OS were was 42.4 months (95% CI, 39.02–45.79) and 44.34 months (95% CI 41.9–46.9) respectively, while both median DFS and OS were not reached, and their conclusion that metronomictherapy with low dose capecitabine was safe and the results need to be compared with control arm in the future studies (Alagizy et al., 2015). 

Notably, several international trials were conducted also in an attempt to improve the treatment outcome in TNBC via an extended period of treatment. Colleoni et al conducted a large phase III study as a part of the larger trial (IBCSG 22-00) where 724 patients with TNBC were subjected to receive metronomic cyclophosphamide 50 mg/day orally and oral methotrexate 2.5 mg/twice days 1 and 2 weekly for 1 year or placebo after finishing the standard adjuvant chemotherapy, they found no significant reduction of DFS in maintenance therapy in the general group, however, they noticed that cases with TNBC with node-positive had better 5 year DFS rate in maintenance group (72.5%) than that non-maintenance group (64.6%) (HR = 0.72; 95% CI 0.49–1.05) (Colleoni et al., 2016). As a part of (IBCSG 22-00) trial also, Pruneri et al found that patients with so-called lymphocyte-predominant TNBC showed a greeter even statistically insignificant benefit from metronomic schedule (Pruneri et al., 2016). The last update of CIBOMA trial presented in the ASCO post by DR Martin about the role of adjuvant capecitabine in TNBCi n the phase III CIBOMA/GEICAM trial, he showed that, in the overall study population, extending adjuvant capecitabine did not improve survival but in a subgroup of non–basal-like TNBC, extending capecitabine treatment demonstrated improvement in both disease-free and overall survival and he suggest that more in-depth characterization of TNBC might help in treatment selection and he said that their study not finished and they will plan to look at the genomic characteristics of disease to identify which molecular subgroups will benefit from the addition of capecitabine (Martín et al., 2019). Unfortunately, the data of the survival results of SYSCBS-001 trial not published till now.

Data from previous studies supported the use of extended adjuvant treatment in TNBC specially the metronomic schedule, although capecitabine was heavily investigated in this researches, its efficacy not truly determined to has a survival benefit. Our results compared to the results of the previous studies showed promising efficacy, however, there were some limitations in our study, the most important one was small sample size and there was no control arm, also, the study lacked the cases subjected to neo-adjuvant therapy. Regarding the tolerability, metronomic docetaxel with the dosing schedule used in our study was tolerable and compliant for patients, however, there is no adequate data in the literature regarding the treatment-related adverse effects of this regimen to be compared with our results.

*Summary and Recommendation*

In the last years, efforts had been done to improve the treatment outcome in TNBC due to the lack of targeted therapy. One of the explored methods is using extended adjuvant chemotherapy especially with mCTH after finishing the primary standard of care treatment. We used in our study a novel approach of metronomic docetaxel as an extended adjuvant approach after the standard adjuvant treatment for patients with early TNBC. The results of our study showed that, extended adjuvant treatment with metronomic docetaxel for patients with operable TNBC has a survival benefits with acceptable treatment-related adverse effects. However, due to the small sample size and lack of control arm, further researches are needed to detect the actual benefit from this novel regimen and this need multicentre randomized control studies with a large number sample size. 
